# Predictive models for personalized asthma attacks based on patient’s biosignals and environmental factors: a systematic review

**DOI:** 10.1186/s12911-021-01704-6

**Published:** 2021-12-09

**Authors:** Eman T. Alharbi, Farrukh Nadeem, Asma Cherif

**Affiliations:** 1 Department of Information Systems, King Abdulaziz University, Jeddah, Saudi Arabia; 2 Department of Information Technology, King Abdulaziz University, Jeddah, Saudi Arabia

**Keywords:** Prediction, Machine learning, Asthma attack, Biosignals, Environmental factor

## Abstract

**Background:**

Asthma is a chronic disease that exacerbates due to various risk factors, including the patient’s biosignals and environmental conditions. It is affecting on average 7% of the world population. Preventing an asthma attack is the main challenge for asthma patients, which requires keeping track of any risk factor that can cause a seizure. Many researchers developed asthma attacks prediction models that used various asthma biosignals and environmental factors. These predictive models can help asthmatic patients predict asthma attacks in advance, and thus preventive measures can be taken. This paper introduces a review of these models to evaluate the used methods, model’s performance, and determine the need to improve research in this field.

**Method:**

A systematic review was conducted for the research articles introducing asthma attack prediction models for children and adults. We searched the PubMed, ScienceDirect, Springer, and IEEE databases from January 2000 to December 2020. The search includes the prediction models that used biosignal, environmental, and both risk factors. The research article’s quality was assessed and scored based on two checklists, the Checklist for critical Appraisal and data extraction for systematic Reviews of prediction Modelling Studies (CHARMS) and the Critical Appraisal Skills Programme clinical prediction rule checklist (CASP). The highest scored articles were selected to review.

**Result:**

From 1068 research articles we reviewed, we found that most of the studies used asthma biosignal factors only for prediction, few of the studies used environmental factors, and limited studies used both of these factors. Fifteen different asthma attack predictive models were selected for this review. we found that most of the studies used traditional prediction methods, like Support Vector Machine and regression. We have identified the pros and cons of the reviewed asthma attack prediction models and propose solutions to advance the studies in this field.

**Conclusion:**

Asthma attack predictive models become more significant when using both patient’s biosignal and environmental factors. There is a lack of utilizing advanced machine learning methods, like deep learning techniques. Besides, there is a need to build smart healthcare systems that provide patients with decision-making systems to identify risk and visualize high-risk regions.

## Background

Asthma is one of the most common chronic respiratory diseases that significantly impact patients and their families. It raises breathing difficulties because it arouses the airways of the lungs and makes them narrow  [1]. The World Health Organization (WHO) has reported that it affects on average 7% of the world population, and it is estimated that the rate of asthma deaths between 2015 and 2030 for the Eastern Mediterranean Region will increase  [2].

The symptoms of asthma disease are coughing, periods of wheezing, chest tightness, and shortness of breath  [3]. These symptoms range from mild to severe from one person to another and may happen frequently or rarely. An asthma attack occurs when these symptoms get worse. There are two categories of risk factors that lead to an asthma attack; patient biosignals and environmental conditions. Many biosignal factors can indicate the possibility of getting an asthma attack, such as lung function, asthma level, smoking status, family history, and clinical data [4]. The environmental factors include weather and air pollution, which are recorded through meteorological stations using multiple sensors. The weather factors that affect asthmatic people include cold, humidity, rain, and wind. In contrast, air pollutions include the small Particulate Matter (PM_2.5_m) with an aerodynamic diameter greater than 2.5 micrometers and PM_10_m with an aerodynamic diameter less than ten micrometers) and ozone (O_3_). The particulate matter comes from cars and factories’ emissions, smoke, and road dust [5–7].

Preventing asthma attacks is the main challenge for asthma patients. It requires keeping track of any risk factor that can cause an attack then predict whether special precautions should be taken to prevent the attack. Real-time monitoring and prediction of asthma attacks are the most promising fields of research  [8, 9]. They help follow up the appropriate mitigation actions to minimize risks.

Recent studies had connected the existence of asthma disease with different features and built various models to predict its existence and exacerbations  [10–13]. Clinical features, patient biosignals, and environmental data can be used to build models for asthma attack prediction.

In this study, we present a systematic review concerning these models. We describe and discuss the existing model’s properties, e.g., population characteristics, data acquisition, predictors, utilized machine learning techniques, and model performance. By understanding these properties, we will provide recommendations for future research applied in asthma attack prediction, supporting improvements and progress in this domain.

The structure of this paper is designed as the following. The related work section discusses some systematic review articles for asthma attack prediction models. In the research strategy, we define the selection criteria for the included articles. In search results, we introduce a detailed explanation for each research article from different aspects. Next, we present a discussion and future research directions. The conclusion is provided in the final section.

## Related work

A variety of surveys have been introduced to review the existing models for asthma prediction. Most of these studies had focused on the models that predict asthma as a disease for children and adults [14–17].

The models included in these reviews were mainly based on using the patient biosignals, parent’s reports, clinical features, and physiological factors (e.g., the historical health record for the parents, lung functions, and the existence of atopic diseases or allergies). The reviewed models were specially designed for use in primary care diagnostic and clinician’s decision-making.

It is worth noting that a few studies have introduced a systematic review of the prediction models designed to predict asthma attack and exacerbation  [15, 16, 18, 19]. The selected models in existing reviews were based on analyzing biosignal/environmental risk factors. However, none of these surveys had discussed the predictive models based on the environmental risk factors in addition to the biosignal risk factors. This review is conducted to answer the following research questions: What is the impact of using one or two different categories of asthma risk factors on the asthma attack prediction model’s performance? Do the existing studies provide reliable and accurate asthma attack prediction models?.

To answer these questions, we investigate the existing research works that propose asthma attack prediction models based on different asthma risk factors as predictors. We analyze these works based on several criteria and compare their performance. In addition, we provide trends in predicting asthma attacks using machine learning, limitations, and future research directions.

## Search strategy

**Fig. 1:**
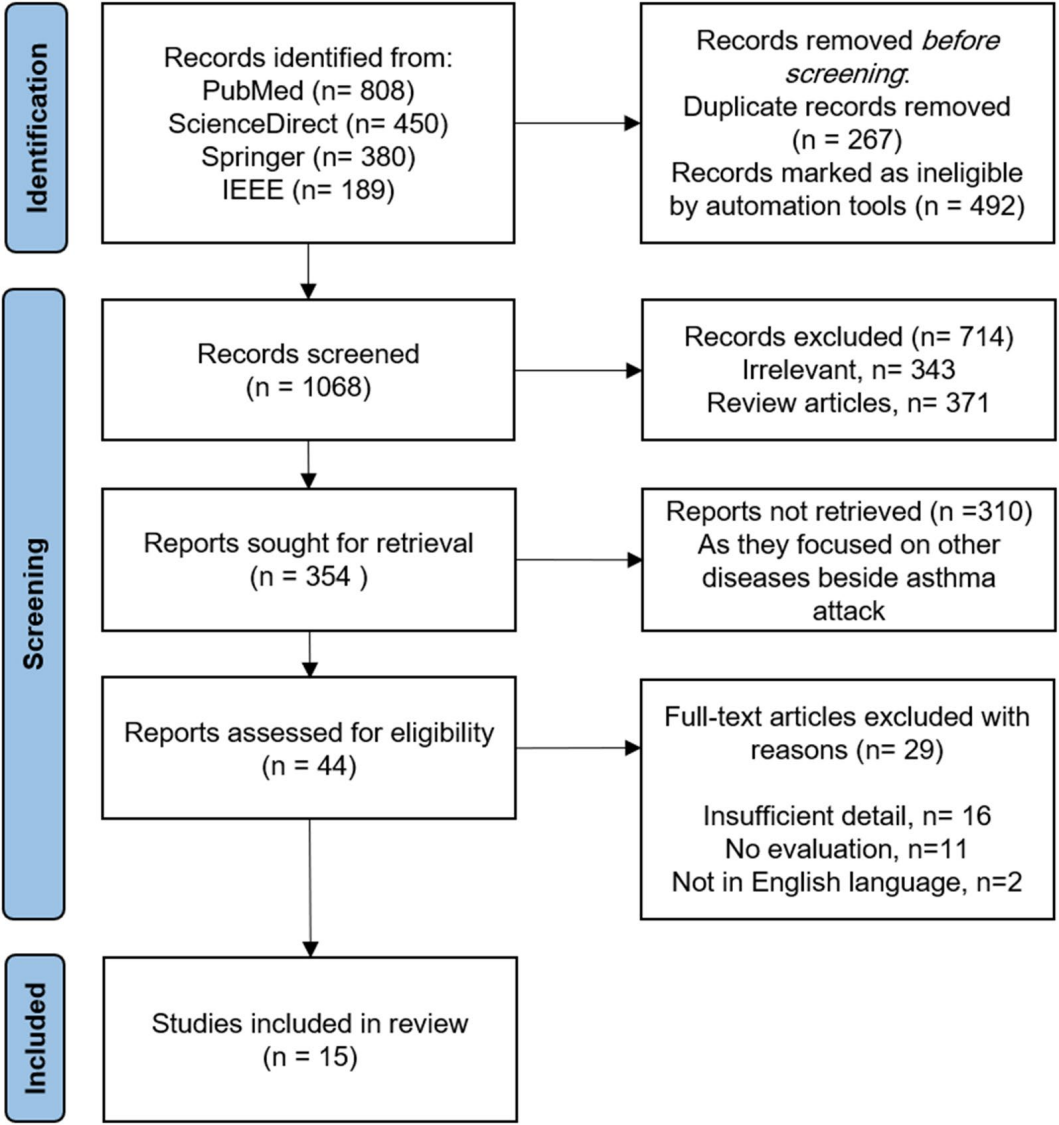
PRISMA flow diagram for the systematic review

In this research, we rely on the updated version of the Preferred Reporting Items for Systematic Reviews and Meta-Analyses (PRISMA) guidelines  [20]. It consists of three main stages, identification, screening, and included, as shown in Fig. 1.

### Search sources and identification

We explored multiple databases to find eligible research articles, the PubMed, ScienceDirect, Springer, and IEEE. The search queries used are: “asthma attack/ exacerbation prediction model”, “predictive model of asthma attack/ exacerbation”, “asthma attack prediction using biosignals data”, “and asthma attack prediction based on environmental factors”. The search yielded 1827 records, 808 from PubMed, 450 from ScienceDirect, 380 from Springer, and 189 from IEEE. 267 duplicate records were removed, and 492 records were marked as ineligible by the automation tool. The remaining records after this elimination were 1068.

### Study screening

**Fig. 2:**
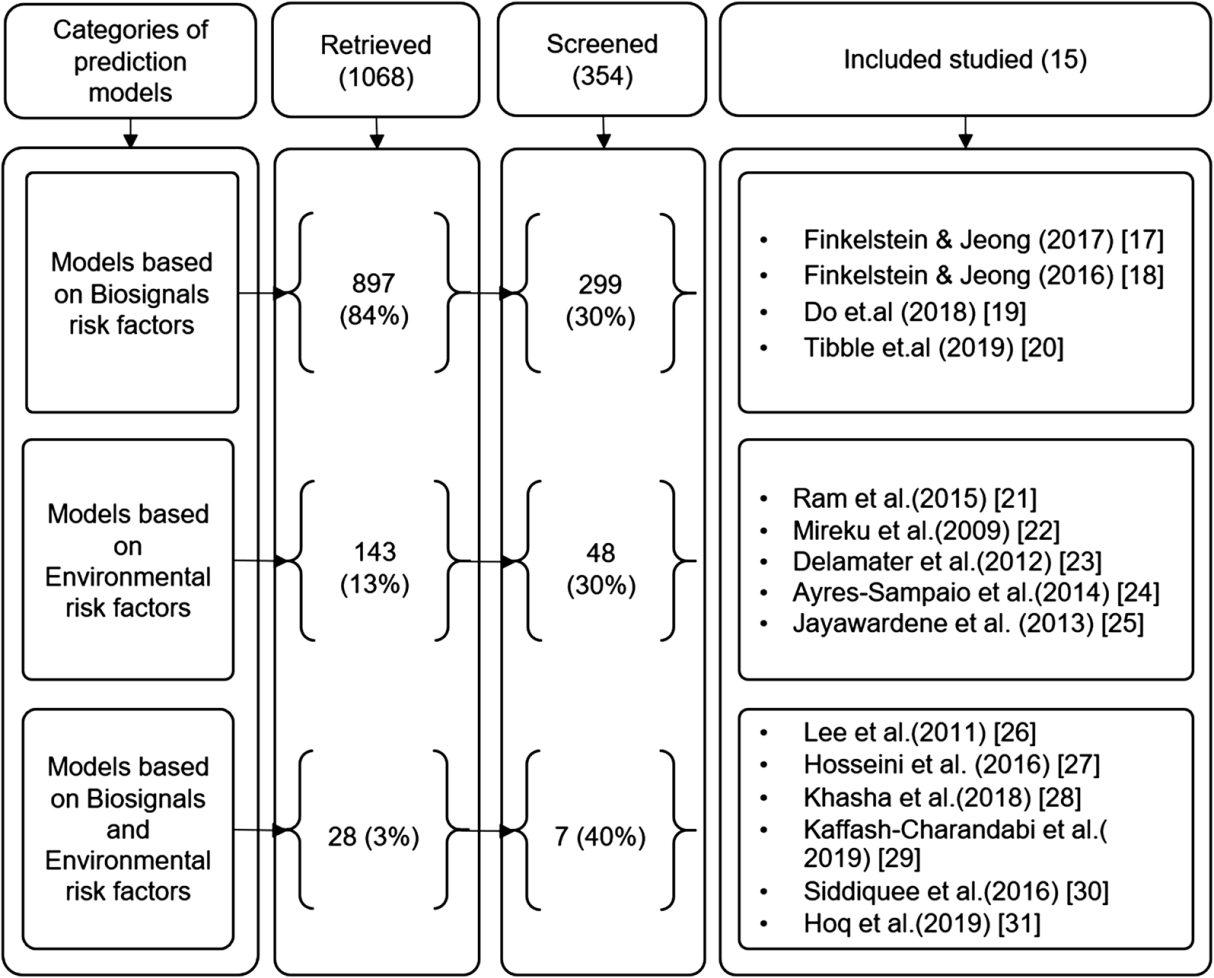
Categories of retrieved studies with the screened percentage and included researches

This survey focuses on the studies that propose asthma attack prediction models. It excludes works providing asthma disease prediction models as they are out of scope. All eligible studies use different asthma triggers in the prediction process. We only considered studies that use biosignals and environmental risk factors. We categorized the retrieved asthma attack prediction models into three categories, models that used biosignals risk factors only, models that used environmental risk factors only, and models that used both risk factors. Most of the retrieved prediction models use only biosignals risk factors (84% of the total articles), followed by the prediction models that only use environmental risk factors(13%). In contrast, the articles that used both of these triggers were very few (3%). We screened 30% of the first two categories and 40% of the last category as these models are our scope of interest (see Fig. 2). The second and third authors of this study screened the titles and abstracts of all retrieved articles. Records marked as ineligible manually were 714. Of these, 343 citations were excluded as they contained irrelevant articles, and 371 were removed as they provided systematic reviews. The remaining articles were 354. The eliminated articles were 310 as they provide a prediction model for other diseases besides the asthma attack, which is not related to our review. The final retrieved full-text relevant articles were 44.

### Included studies

Each of the 44 retrieved full-text articles was screened and evaluated independently by the main author of this study. Two different checklists were used to assess these papers based on their contents; the Checklist for critical Appraisal and data extraction for systematic Reviews of prediction Modelling Studies (CHARMS)  [21] and the Critical Appraisal Skills Programme clinical prediction rule checklist (CASP)  [22]. These checklists were designed especially for assessing the aspect of predictive modeling articles. The abstract was reviewed accordingly with the CHARMS checklist, as shown in Table 1. Then the body was assessed according to the CASP checklist, as illustrated in Table 2. The assigned scores were used to obtain a final inclusion judgment. The articles which got the lowest scores were excluded. Indeed, they either present inadequate details (16 articles), or have no clear evaluation of their models (11 articles), or are Non-English study (2 articles). We found only fifteen articles that met our inclusion criteria of current study. The authors did not obtain unpublished related articles.

**Table 1:** CHARMS checklist for assessing the abstracts of selected research articles [22]

	Abstract selection criteria	Criteria description
1	“Type of prediction model”	A predictive model to predict the expected event of Asthma attack
2	“Intended scope”	To monitor asthma patients, make decisions on the need for therapy, and help prevent asthma attacks
3	“Type of prediction modeling studies”	Model development researches in which a previously generated model was improved
4	“Target population”	All age groups
5	“Outcome to be predicted”	Asthma attack or exacerbation, asthma risk level, the cause of the asthma attack
6	“Timespan”	Predictors measured between 1–2 weeks / < 1 month; outcomes measured directly after data acquisition
7	“Intended moment of using the model”	Preventing asthma attacks and recognizing its exacerbation

**Table 2:** CASP checklist for assessing the body of the selected research articles [29]

	CASP questions
Q1	Is there a clear definition of the prediction model?
Q2	Does the dataset used to build predictive models sufficiently represent of the patient population?
Q3	Was the predictive model tested on a diverse patient group?
Q4	Were the outcome and the predictor variables examined in a blinded manner?
Q5	Were the evaluation of the outcome and the predictor variables performed on the whole sample that was initially selected?
Q6	Are the statistical approaches that were utilized to build and validate the predictive model described in detail?

## Search results

**Fig. 3:**
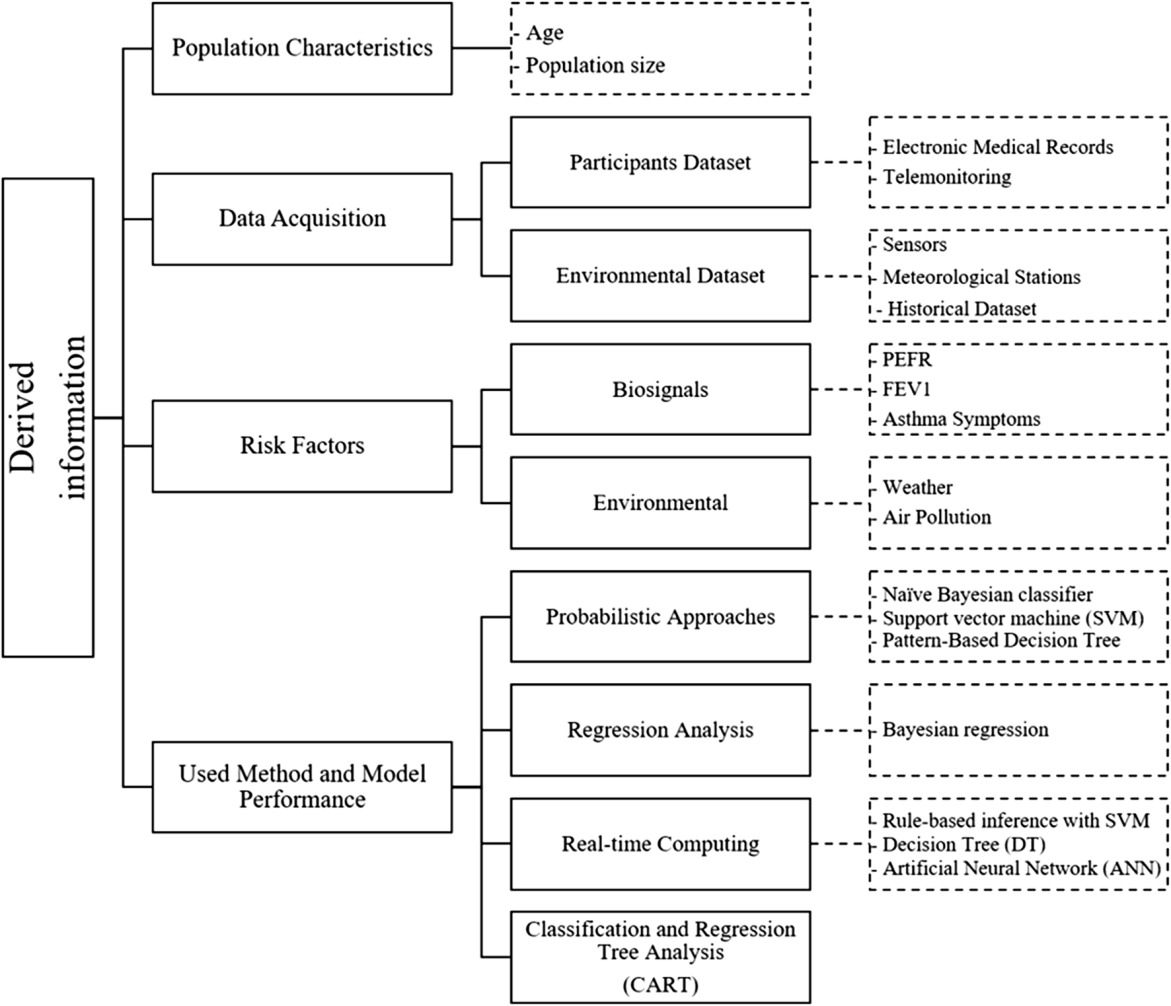
Taxonomy of the review output

Each selected model is reviewed descriptively. In the upcoming subsections, we explain the collected information that covered the population characteristics, data acquisition method, used risk factors, development method, and the model performance reported in the derivation cohorts such as specificity, sensitivity, precision, and accuracy ratios. At the end of this section, we placed Fig. 3 to present the review summary taxonomy that shows the derived information with different attributes.

### Population characteristics

The designed models were developed to predict asthma attacks for children and adults. However, the validation of these models was tested on adults only, but it can be used for children. The population size varied from 1 [23] to 500,000 [24] participants. However, having a limited number of participants in a prediction study, such as [23, 25], restricts the circumstances of the study and leads to unreliable results. Some studies used a large population size [26–28] and acquired their data from national databases; therefore, some participants were excluded due to uncompleted records.

### Data acquisition

The used dataset in the existing prediction models included data from two primary sources, biosignals and environmental. Each of these datasets was acquired by different techniques. This section introduces the used methods in the existing studies for each data source. Table 3. introduces a comparison to summarize the population size and the data acquisition methods for all the included studies.

#### Participant’s datasets

Most of the models were built using home-based telemonitoring technology, which was increasingly used recently to monitor and control different diseases, including asthma. This method was used in [25, 29–32], where the patients record their symptoms daily and submit them through the application interface. This acquisition method relies on the patients’ ability to use technology and increases the burden on them. Other studies regarded patients who had initially visit the emergency department [26–28, 33]. However, research on different groups and populations is required to generalize the model outcomes and to confirm the study findings. This concern was solved by Tibble et al. [24] through utilizing a historical dataset of the Asthma Learning Healthcare System (ALHS), rising with the most significant sample size of all the covered studies (n = 500.000). Besides, Jayawardene et al. [34] collected the patient’s data within 49 countries by using Electronic Medical Record (EMR), including the patient’s symptoms, physical examination, and lab tests.

Hosseini et al. [23] developed another method by employing a smartwatch application to record the biosignals data through a built-in wireless sensor, which gives a better way to build a personalized prediction model. The Do et al.  [35] study raised significant concerns since it depends only on a randomly generated dataset, which can be very unpredictable and unreliable.

#### Environmental datasets

Most of the studies acquired the environmental data from meteorological agencies and national air pollution monitoring stations [23, 31, 36].

The other method used to acquire these data was by using built-in or networked sensors. Siddiquee et al. [37] proposed a system built on a network of sensors that can collect different environmental data and analyze them through the Internet of Things (IoT) sensors. On the other hand, Hoq et al. [32] proposed a framework that utilized a small sensor that can be carried by the user and connected to his mobile via Bluetooth to transfer the sensed data. However, using a biosignal sensor to monitor air pollutions is not practical and difficult to deploy. Moreover, these sensors may not be accurate and do not give correct measurements as the agency stations do.

**Table 3:** Comparison of the reviewed articles regarding the population size and data acquisition methods

References	Population size	Data Acquisition method
[29]	7001 records, 350 patients	Telemonitoring data
[30]	3470 records, 174 patients	Telemonitoring data
[35]	Random generated dataset with size 130,000 text	Randomly generated text
[24]	Asthma Learning Healthcare System (ALHS) dataset includes 500,000 individuals	Historical data
[33]	50 ED visits, 3768 tweets	Twitter data, Google search interests, and ED data from hospital
[26]	25,401	ED visits Historical data+ Health records
[27]	100,000	ED visits
[28]	18,409	ED visits
[34]	168,825	EMR
[31]	33 participants	Historical data+ MS
[23]	1 participant	Physiological and environmental wireless sensors
[36]	32 participants	Historical data+ MS
[25]	3 participants in 80 different situations	Historical data+ MS
[37]	–	IoT Sensors
[32]	–	Personal sensor

### Predictors

All the predictors used in the prediction models have evidence of being linked to asthma attacks and exacerbation. These predictors are divided into two main categories, biosignal and environmental risk factors. Most researchers used one type of risk factor in the prediction process, ignoring the importance of other factors. Few researchers introduced an asthma attack prediction model using both biosignal and environmental factors.

The included articles were separated into three categories based on the used predictive features. The first research articles category covers the studies that use biosignals data only to build their prediction models [24, 29, 30, 35]. The second research articles category includes studies that use environmental data only to build their prediction models [26–28, 33, 34]. Finally, the third category includes the studies that used both biosignals and environmental data in their models [23, 25, 31, 32, 36, 37].

In the following, we discuss each of these categories then summarize our findings in Table 4.

#### Predictive models based on the patient’s biosignal risk factors

Peak Expiratory Flow Rate (PEFR) and Forced Expiratory Volume in 1 second (FEV1) are the most common biosignal factors used to measure breathing stability  [38]. PEFR variable was mainly used in seven out of the fifteen studies [23–25, 29–31, 36] to predict asthma attacks. Other biosignal factors were combined with PEFR to explore the utility of using additional attributes to increase the reliability of the prediction models.

Finkelstein and Jeong [29, 30] employed physical activity, respiratory infections, used medications, colds, and sleep disorders. Asthma severity, smoking Status, blood eosinophil count, obesity, comorbidity, previous medication usage, and history of asthma attacks were applied beside PEFR in the prediction model introduced by Tibble et al. [24]. Besides all the previous factors, Lee et al. [31] applied extra attributes, including food allergy, dermatitis, rhinitis, and conjunctivitis.

On the other hand, FEV1 was used in two studies only. Hosseini et al. [23] introduced their prediction model based on FEV1 and PEFR without considering any other biosignal factors. In contrast, Do et al. [35] used FEV1 with lung functions, night awaking, used medication, activities, and symptoms.

According to these studies, using additional biosignal asthma risk factors significantly improve model performance. In Lee et al. [31], who utilized the highest number of risk factors, the model achieved a high accuracy of 0.87 and a high recall of 0.85. In comparison, the model built by Hosseini et al. [23], which utilized two factors only, achieved less accuracy of 0.80. Thus, the model performed better when using multiple attributes. The accuracy of the other models [29, 30, 36] falls between these two percentages. One exception is Kaffash-Charndabi et al. [25] predictive model, which used only the PEFR as a biosignal risk factor and got an accuracy of 0.93. However, this result comes out due to testing the model on three participants only, which makes it unreliable regarding the performance of the other models, which were trained and tested with a large dataset.

#### Predictive models based on the environmental risk factors

Asthma is highly associated with environmental factors. A large body of research articles were promoting the correlation between disclosure to weather and air pollution with asthma attacks [6, 7, 39, 40]. Various models were introduced to predict asthma attacks based on analyzing weather conditions and air pollution concentration. These studies’ prediction target was the number of asthma patients’ visits to the emergency department (ED). Temperature (T), humidity (H), nitrogen dioxide (NO2), sulfur dioxide (SO2), ozone (O3), carbon monoxide (CO), particulate matter (PM10), and particulate matter (PM2.5) were the most prevalent weather and air pollution factors employed in the studies  [26–28, 33].

Ram et al. [33] used Twitter data and Google search interests besides these environmental factors for asthma attack prediction. Using such datasets provides a better opportunity for early prediction of asthma risk and can prevent the attack. However, the model performance gets lower when using these data, with an accuracy of 0.70 and 0.89 without these data. Barometric pressure (BP) was one of the weather factors tested by Mireku et al. [26] to determine its effect on increasing asthma symptoms besides all the previous variables. The model revealed that BP has no effect on asthma patients and did not change the prediction result.

Delamater et al. [27] had tested each of the previously mentioned factors and found that the increase in CO, NO^2^, and PM_2.5_ were significantly impacted the rate of asthma hospitalization with a positive coefficient correlation of 0.79, 0.79, 0.93, respectively. In contrast, T and H produced mixed results, and O_3_ was non-significant with a relationship of 0.03. Vegetation density (VD) was applied as a new predictor in the model introduced by Ayres-Sampaio et al. [28]. The outcomes of the study show that VD has consistent relationships with asthma with 0.6 Pearson correlation coefficients. The result illustrates that people with asthma are at high risk of getting asthma attacks in the urbanized area and sparsely vegetated areas, leading to hospital admissions.

The result of these studies leads other researchers to the most harmful air pollution factors to indicate them in future studies. However, the studies lack in using individual data and variations in the demographic structure of the population, which limits the ability to establish a causal link between the change in environmental factors and asthma outcomes.

#### Predictive models based on the biosignal and environmental risk factors

Predicting the asthma attack becomes more accurate and gives better performance when using both of the risk factors, the biosignal and environmental. Lee et al. [31] introduced a study that shows how to contribute to the model performance by applying multiple predictors from different sources. They used 37 attributes; 19 for the patient biosignals, 14 for air pollution, and 4 for the weather. The model’s performance demonstrates that it can predict asthma attacks with an accuracy of 0.87.

Other studies [23, 36] considered limited biosignal attributes and focused on applying more environmental data, including air quality index (AQI), weather data, and traffic. Their model’s performance was under 0.80 accuracy, which could be improved by applying additional biosignals data. Kaffash-Charandabi et al. [25] developed another study based on using PEFR and the patient’s medical history. The limitation of this study was the limited number of its participants, as they were only three participants who record their data in different situations.

Two studies built a prediction framework with a new interpretation of the situation of concern in this review. Hoq et al. [32] and Siddiquee et al. [37] proposed a smart framework to predict asthma attacks by deploying wireless sensors to acquire biosignals and environmental data. However, the proposed structures were limit these data to a few risk factors such as food allergy, humidity, CO, and NO^2^, which is not clear how the model would perform with these factors in the real world.

**Table 4:** Comparison of the reviewed articles regarding the used risk factors

References		Risk factors	
	Personal	Weather	Air pollution
[29, 30]	PEFR, respiratory symptoms, presence of cold, sleep disturbances, medication, and physical activity	–	–
[35]	FEV1, Lung functions, night awaking, used medication, activities, and symptoms	–	–
[24]	Asthma severity, smoking status, PEFR, asthma attack history, medication, obesity, comorbidity, and blood eosinophil count	–	–
[33]	Number of ED visits	–	PM_2.5_, O_3_, CO, and NO^2^
[26]	Number of ED visits	Temperature, humidity, and barometric pressure	–
[27]	Number of ED visits	Humidity and temperature	PM_2.5_, CO, O_3_, NO^2^, PM_10_
[28]	Number of ED visits	Temperature and humidity	NO^2^ and Vegetation density
[34]	Demographic data, number of asthmatic children in the selected schools, and the used medications	Temperature	PM_2.5_, PM_10_, SO^2^, NO^2^, CO, and O_3_
[31]	PEFR, Nose symptom, eye symptom, skin symptom, night symptom, day symptom, fever, dermatitis, rhinitis, asthma medicine instructions, conjunctivitis	Temperature, absolute maximum and minimum temperature, and humidity	HydraCarbon,HydraCarbon2, PH, PM_10_ CO, SO^2^, O_3_, NO^2^
[23]	PEFR and FEV 1	Temperature, precipitation intensity, wind speed, humidity, pressure, and visibility	SO^2^, PM_2.5_, CO, NO^2^, and PM_10_
[36]	PEFR and patients’ location	Humidity, absolute maximum and minimum temperatures, average temperature, pressure, rainfall, and wind.	O_3_, SO^2^, NO^2^, CO, PM_10_
[25]	PEFR, location, and medical history.	Temperature, barometric pressure, and humidity	PM_10_, NO^2^, CO, O_3_, SO^2^
[37]	Food allergen	Humidity	Pollen
[32]	Medication plan	–	CO, NO, dust and smoke

### Development method and model performance

There were four unique model development methodologies specified in the reviewed research articles: Probabilistic Approach, Classification and Regression Tree Analysis (CART), Regression Analysis, and Real-Time Analysis. Table 5 presents details for the used methods with the performance of each model.

#### Probabilistic approach

The probabilistic approach is based on the probability theory. It consolidates probability distributions and random variables into a model of phenomenon or events and gives a probability distribution as a solution [41]. A probabilistic approach was used in four of the analyzed research articles to build the prediction model [29, 31, 34, 35].

Finkelstein and Jeong [29] and Lee et al. [31] used machine learning to create a model that assigned a high or low probability of asthma exacerbation. They used roughly 70% of the data for model building and 30% for verification. The models were evaluated using different performance matrices. These matrices were introduced for the model as a unit but not to particular values for development and verification. Finkelstein and Jeong [29] used eight-day lag inputs from a telemonitoring system as predictors. A support vector machine (SVM), an adaptive Bayesian network (ABN), and a naive Bayesian classifier (NB) were utilized. This study found that the ABN classifier has higher sensitivity and specificity (1.00, 1.00) than SVM (sensitivity 0.84 and specificity 0.80), and NB classifier (sensitivity 0.80, specificity 0.77). Do et al. [35] built a model to classify the asthma attack into three categories. They trained their model with Bag of Words and tested it with a random text using Tensorflow Text Classification (TC) result in an accuracy of 0.98.

Jayawardene et al. [34] used a generalized estimating Equation (GEE) to predict asthma attacks. They used a cut-off value estimated by a 3-year mean value of seizures per day in the period between 2008 -2010. The highest asthma attacks are found in the fall and summer, and the lowest are found in spring and winter.

Lee et al. [31] used a five-day lag to train the classifier with the dataset acquired from EMR and environmental sensors. They applied Pattern-Based Class-Association Rule (PBCAR) and Pattern-Based Decision Tree (PBDT) and compared their results. The experimental results show that PBDT outperformed PBCAR with a slight difference, which delivers an accuracy of 0.87 and 0.86, respectively.

#### Classification and regression tree analysis (CART)

CART analysis is unlike traditional data analysis methods. It is a tree-building technique that is effective for creating clinical decision rules [42]. In addition, CART can reveal complicated interactions between predictors that may be difficult to discover using traditional multivariate techniques.

Finkelstein and Jeong [30] developed an asthma exacerbation prediction model one day ahead of time based on the preceding 7-day lag by using the CART algorithm. Telemonitoring data was used in the development and verification of the model. Three evaluation models were conducted, and each model predicts whether the asthma is “Normal” or “Exacerbation” for the next day. The resulting algorithm had a specificity of 0.97, a sensitivity of 0.64, and an accuracy of 0.80.

#### Regression analysis

Regression is a statistical measure used to determine the relationship between independent and dependent parameters [43].

Different categories of regressing were used in three of the analyzed research articles [26–28]. These studies introduced different models to determine the probability of increasing asthma ED visits according to weather or air pollution concentration changes. Mireku et al. [26] used Bayesian regression and found that with two to three days lag, the increase in humidity and temperature cause a rise with 1.8 additional asthma ED visits.

Delamater et al. [27] built multiple models to determine the relationship between environmental variables and asthma ED visits. Bayesian regression was used with temporal random effects, which improved model fit and produced better accurate and precise predictions.

Ayres-Sampaio et al. [28] applied Land-Use regression (LUR) results with a consistent relationship between T, NO2, and VD with asthma in all seasons. T and NO2 were positively associated with the admissions where Pearson correlation coefficients ranged from 0.351 to 0.600, 0.405 to 0.513, respectively. VD was negatively related to the ED admission where the coefficients ranged from 0.376 to 0.498. Also, H had a high relationship where the ratio ranged from 0.54 to 0.81.

#### Real-time computing

Real-time computing is based on recognizing and performing different functions within a predictable time and storing a large amount of data acquired from the controlled system [44].

There were four works [23, 25, 33, 36] that used real-time computing to predict asthma attacks per individual. Different wireless sensors were employed to acquire data from users and the environment. Hosseini et al. [23] developed a model to divide the risk of having an asthma episode into three categories (low, medium, and high risk) using a random forest classifier with 10-fold cross-validation. The biosignals data were recorded through a built-in smartwatch wireless sensor, while the environmental data were acquired from different meteorological wireless sensors. Data were analyzed in real-time through the cloud platform. The prediction model could classify the risk level with an accuracy of 0.80.

Kaffash-Charandabi et al. [25] introduced another real-time prediction model based on multiple reasoning mechanisms for a more reliable model. Rule-based inference with SVM was used to predict asthma attacks for each individual and performed an average accuracy of 0.93. the model was trained and tested on three participants only, and it is not clear how the model would perform when tested on more populations.

Ram et al. [33] developed a real-time prediction model for asthma ED visits based on Twitter, Google search data, and environmental variables. They examined different techniques to validate the model include Decision Tree (DT), Artificial Neural Network (ANN), and a combination of both (DT+ANN). The result of using ANN alone was the same as using both techniques and its surpassed DT technique, resulting in an accuracy of 0.70 and 0.67, respectively, which considered low and could be enhanced by using different machine learning techniques or more specified dataset.

Another comparison was performed by Khasha et al. [36] between six different methods. Also, DT had better performance and surpassed LR, RF, SVM, and gradient boosting (GB). The study used different measurements like sensitivity, specificity, F-measure, G-means, accuracy, and precision. A weakness of this study was that it fails to represent the output of these measurements with percentages or numbers, and it used only figures to describe the outcomes. Two more articles [32, 37] also proposed a prediction model based on real-time analysis. The proposed frameworks are built based on activating multiple wireless sensors to acquire real-time data from the user and his surrounded environment. However, using a biosignal sensor to monitor air pollutions is not practical and challenging to employ.

**Table 5:** Comparison of the reviewed articles regarding the used development method and model performance (the used symbols SN: sensitivity, SP: specificity, AC: accuracy)

References	Development method/machine learning technique	Model performance
[29]	NB, ABN, and SVM	SN: 80%, 100%, 84%; SP:77%, 100%, 80%; AC: 77%, 100%, 80%
[30]	CART	SN: 64%, SP: 97%, AC: 80%
[35]	TC	AC: 97%
[24]	NB, SVM, and random forests	–
[33]	DT, ANN, and DT+ANN	AC: 65.18%, 66.25%, 66.25% respectively
[26]	Bayesian regression	Increase in T ->1.8 ED visit Increase in H ->1 ED visit
[27]	LR	Coefficient correlation of 0.79 (CO), 0.79 (NO2), 0.93 (PM2.5), and 0.03 (O3)
[28]	LR	Pearson correlation coefficient: 0.60
[34]	GEE	Probability 50% of an asthma attack with low T and high AP
[31]	PBCAR and PBDT	AC: 86.89% and 87.52%, Recall: 84.12%, and 85.59% respectively
[23]	Random forest	AC: 80%
[36]	LR, DT, ANN, SVM, gradient boosting, and random forests	AC, SN, SP, G-mean, precision, and F-measure
[25]	SVM	AC: 93.55%

## Discussion and future research direction

There are numerous limitations to the existing predictive models for asthma attacks (see Table 6). To overcome this, we define some recommendations and propose different solutions to advance the studies in this field.

**Table 6:** Comparison of the the reviewed articles regarding their Pros and Cons

References	Pros	Cons
[29, 30]	Number of participants is adequate The performance of the model is provided with different measurements	Compared different machine learning techniques The used data are not enough to predict asthma attacks Population is not generalized Environmental risk factors were not considered
[35]	Number of datasets is adequate Model accuracy is high	Some personal risk factors were ignored Environmental risk factors were not considered
[24]	Used exhaustive dataset Compared different machine learning techniques	The study proposed only a protocol without the results of the prediction process
[33]	Applied real-time dataset Compared different machine learning techniques	The tweets were taken from the English language only The ED visits data were taken from one hospital only The accuracy of the model is low
[26]	Used sufficient dataset	Air pollution was not considered
[27]	Dataset has many records	Data was lack of patient demographic and residential location information
[28]	Used sufficient dataset	Air pollution factors were limited
[34]	Dataset has many records	Weather factors were limited
[31]	Used exhaustive personal and environmental predictors Compared two different machine learning techniques Increased the model performance by applying a feature selection algorithm	Bio-signals were daily recorded by users The interpretation of the study is difficult for users No services after prediction
[23]	Used exhaustive environmental factors	The prediction result is only three states, without any suggestions for treatment or precautions
[36]	Used exhaustive environmental factors Compared different machine learning techniques The performance of the model is provided with different measurements	Patient medical history and bio-signals were not used Validation results were not expressed by numbers
[25]	Model accuracy is high Map of polluted and safe areas was introduced	Study was performed on three participants only
[37]	A complete framework was introduced to predict attacks, alarm used, and view polluted sites	The model is based on environmental data only It is a proposed framework only
[32]	The proposed framework considering real-time prediction and warning users with risks	Weather factors were not considered Personal sensor does not give an accurate reading Difficult to employ

### Use of multiple risk factors

Most existing asthma attack prediction models use limited risk factors. Large numbers of research articles utilize only biosignals data and ignore the environmental factors. Also, using the telemonitoring system in collecting biosignals data is not an effective method since data is recorded manually by the patients themselves.

In practice, Electronic Medical Record (EMR) represents a better data source as it is much more consistent and robust than telemonitoring data. Also, EMR often includes additional variables observed by the physician. Combining EMR and the wireless sensor is the ultimate method that provides the best historical data and precise biosignals measurements for more accurate prediction results. It is essential to devise new prediction systems that intelligently take the full potential of EMR to collect and update patient data.

### Introducing personalized prediction and prevention models

Most existing asthma attack prediction models were established for clinical use to facilitate the disease diagnosis and decision-making for the physician. Unfortunately, this has led to design models that are unsuitable for personals usage. Indeed, asthma patients need to follow up on all the asthma symptoms and risk factors to avoid sudden attacks. So far, most current models make predictions to identify the ED visits or find the relationship between the attack and different risk factors, ignoring the need for personalized asthma attack prediction and the required precautions that should be followed.

To improve the lives of asthma patients, a prediction model for asthma attacks must immediately alert the user to any unfavorable scenario when a risk threatens the patient before worsening his/her health condition. Ideally, the model should alarm the user with a real-time update for air pollution concentration and climate changes as additional services.

### Improving prediction accuracy

To provide a valuable asthma attack predictive model, it is crucial to provide high sensitivity and accuracy. Indeed, high sensitivity guarantees the model’s ability to classify most patients who will get an asthma attack according to the surrounding risk factors. In contrast, high accuracy means that a patient having a prediction of high risk is undoubtedly expected to have an asthma attack.

The majority of the predictive models examined in this research had an accuracy of 0.87 or below, with two exceptions that did not use a suitable dataset. Besides, sensitivity was not reported for most of the models. Improving the accuracy of asthma attack prediction models without affecting computation complexity is still a work in progress. To the best of our knowledge, no such model can now achieve efficiency levels sufficient for individual use. The model’s accuracy can be improved by combining large datasets consisting of a comprehensive set of relevant factors with advanced machine learning approaches.

With rare exceptions [24, 34] asthma attack prediction models that are currently available utilized small datasets (less than 350 patients). Generally, the accuracy of prediction model improves as the training dataset becomes more comprehensive and the model utilizes multiple predictors. There are a variety of asthma attack risk factors identified through different studies and researches [3, 5, 7, 45]. However, with one exception [25], most existing asthma attack prediction models used less than ten variables. By utilizing a comprehensive set of predictors linked with a large number of populations, the model’s accuracy will further likely improve.

## Conclusion

Establishing a self-management tool for asthma control and attack prediction is still an attractive area for researchers. By reviewing the studies and research in asthma attack prediction models, it can be stated that majority of these studies focused on using specific factors and neglected other essential factors. Asthma prediction requires the use of various risk factors, including biosignals and environmental conditions. Numerous studies introduce a linkage between particular variables and asthma exacerbation, and such articles can be used in the predictor selection process. Moreover, most of the current prediction models do not deliver services to patients. Subsequently, there is a limitation in developing a smart monitoring system of environmental variables that can provide a personalized map with the concentration of surrounding air pollution. It’s essential to build a smart healthcare system that can predict asthma attacks and visualize environmental risks for asthma patients. There is a need to consider all the categories of risk factors with various variables as much as possible to deliver an accurate model. Patients must have access to a decision-making system that alerts them by detecting risk factors and high-risk areas. A dynamic visualizing map for air quality is also required to allow citizens and policymakers to take quick action and avoid polluted areas.

## Abbreviations

WHO: World Health Organization
ED: Emergency Department
EMR: Electronic Medical Record
CHARMS: Checklist for critical Appraisal and data extraction for systematic Reviews of prediction Modelling Studies
CASP: Critical Appraisal Skills Programme clinical prediction rule checklist
PEFR: Peak Expiratory Flow Rate
FEV1: Forced Expiratory Volume in 1 second
AQI: Air Quality Index
CART: Classification and Regression Tree Analysis
GB: Gradient Boosting
SVM: Support Vector Machine
LR: Logistic Regression
ANN: Artificial Neural Network
NB: Naïve Bayesian
ABN: Adaptive Bayesian Network
PBDT: Pattern-Based Decision Tree
PBCAR: Pattern-Based Class-Association Rule
ROC: Receiver operator characteristic
AUC: Area Under Curve
